# Supplementary material for: Facile Electrodeposition of NiCo2O4 Nanosheets on Porous Carbonized Wood for Wood-Derived Asymmetric Supercapacitors
Source: Polymers (Basel). 2022 Jun 21;14(13):2521. doi: 10.3390/polym14132521 (PMC9269009; doi:10.3390/polym14132521)
Supplement: Supplementary file 1 [file polymers-14-02521-s001.zip › polymers-1688260-supplementary.pdf]

Supplementary Information for:

# Facile electrodeposition of $NiCo_2O_4$ nanosheets on porous carbonized wood for wood-derived asymmetric supercapacitors.

**Jingjiang Yang [1], Huiling Li [1], Shuijian He [1,*], Haijuan Du [2], Kunming Liu [3], Chunmei Zhang [4,*] and Shaohua Jiang [1,*]**

[1] Jiangsu Co-Innovation Center of Efficient Processing and Utilization of Forest Resources, International Innovation Center for Forest Chemicals and Materials, Nanjing Forestry University, Nanjing 210037, China; yjj10101010@163.com (J.Y.), yyb23232323@163.com (H.L.)

[2] College of Textiles, Zhongyuan University of Technology, Zhengzhou 450007, China; duhaijuan2009@126.com

[3] Faculty of Materials Metallurgy and Chemistry, Jiangxi University of Science and Technology, Ganzhou 341000, China; liukunming@jxust.edu.cn

[4] Institute of Materials Science and Devices, School of Materials Science and Engineering, Suzhou University of Science and Technology, Suzhou 215009, China; cmzhang@usts.edu.cn (C.Z.)

* Correspondence: shuijianhe@njfu.edu.cn (S.H.); cmzhang@usts.edu.cn (C.Z.); shaohua.jiang@njfu.edu.cn (S.J.)

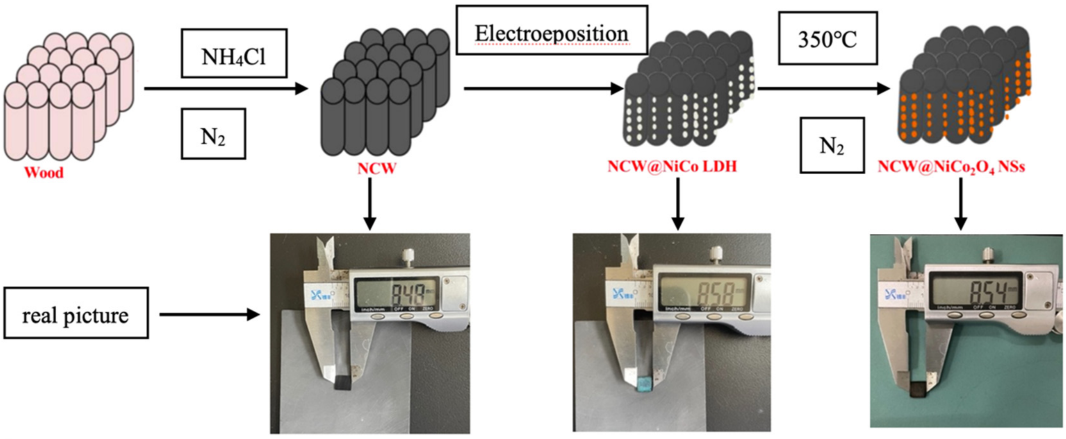


**Figure S1.** Schematic of the synthesis of NCW@$NiCo_2O_4$.

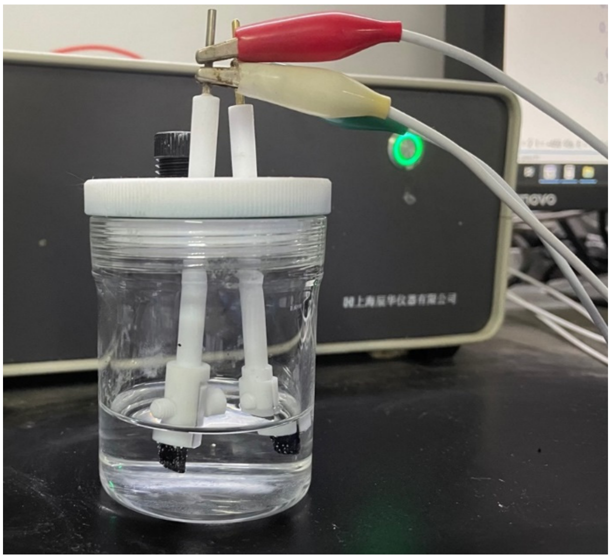

**Figure S2.** Schematic diagram of the electrochemical performance test of the device.

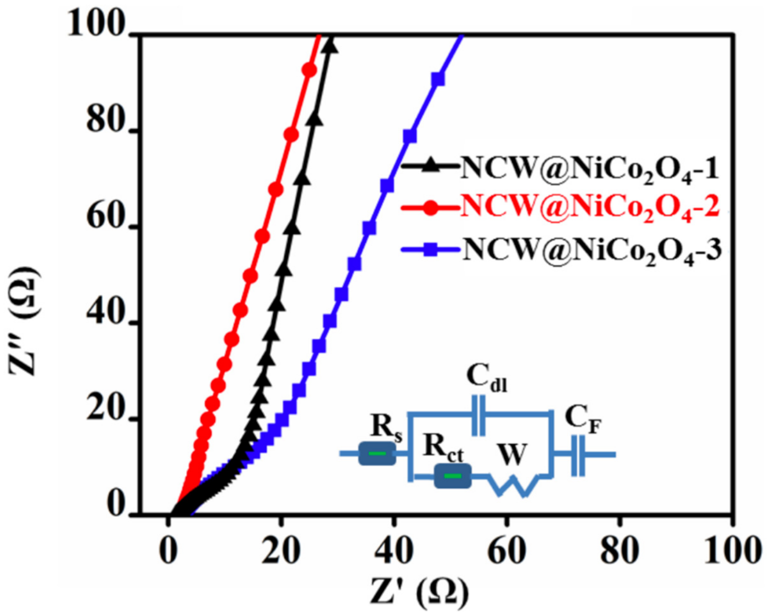


**Figure S3.** Electrochemical impedance spectra of NCW@Ni$Co_2O_4$-x composite electrodes in a certain frequency range (0.01 Hz-10 kHz).

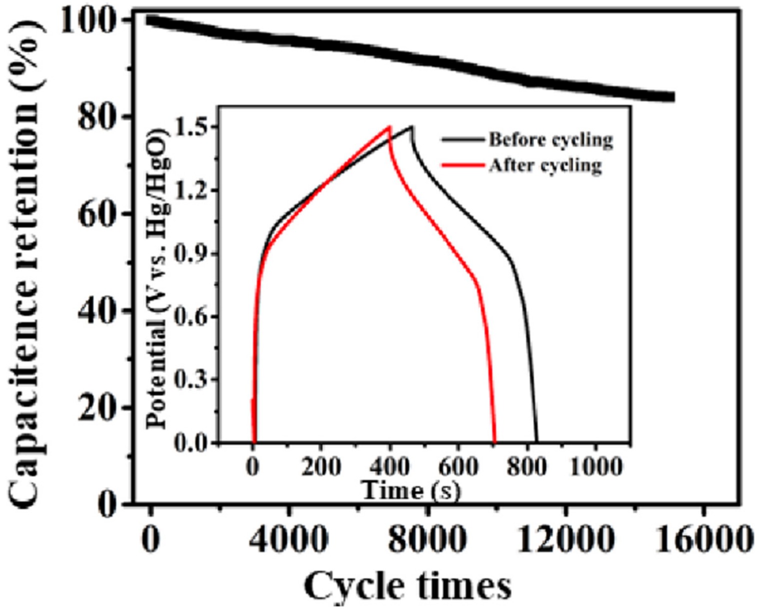


**Figure S4.** Cycling performance of NCW//NCW@Ni$Co_2O_4$ at 10 A $g^{-1}$.

**Table S1.** Mass loading of active material.

| Sample | NCW (mg) | $NiCo_2O_4$ NSs (mg) |
|---|---|---|
| NCW | 10.0 | 0 |
| NCW@$NiCo_2O_4$-1 | 10.0 | 1.0 |
| NCW@$NiCo_2O_4$-2 | 10.0 | 2.0 |
| NCW@$NiCo_2O_4$-3 | 10.0 | 3.0 |
